# Influencing factors of physical exercise behavior among Chinese female university students: a configurational analysis based on fsQCA

**DOI:** 10.3389/fpubh.2025.1695542

**Published:** 2025-10-23

**Authors:** Peng Jia, Delu Meng, Ting Zhang, Shuqi Sun, Xinqi He

**Affiliations:** School of Physical Education, Jiangxi Normal University, Nanchang, Jiangxi, China

**Keywords:** Chinese female university students, physical activity behavior, influencing factors, fsQCA, configurational analysis

## Abstract

**Objective:**

This study investigates how combinations of factors lead to high physical activity levels among Chinese female university students.

**Methods:**

Grounded in Social Cognitive Theory (SCT), we developed an “Individual-Behavior-Environment” triadic reciprocity framework. Using fuzzy-set Qualitative Comparative Analysis (fsQCA), we performed a configurational analysis of physical activity behaviors among 115 Chinese female university students. The study assessed six antecedent conditions across three dimensions: capability cognition (exercise self-efficacy, perceived value of exercise), motivational drive (exercise motivation, subjective exercise experience), and environmental interaction (friend support, social anxiety).

**Results:**

None of the six individual conditions were necessary for high physical activity behavior. Instead, high activity levels emerged from synergistic interactions among multiple conditions. We identified eight distinct equifinal configurations leading to high physical activity, with an overall solution consistency of 0.888 and coverage of 0.722, indicating strong model robustness. These configurations formed four typical driving modes: Immersive Enjoyment, Autonomous Drive, Dual Core Reinforcement, and Efficacy Anxiety Compensation.

**Conclusion:**

This study confirms the characteristics of “multiple conjunctural causation” and “causal asymmetry” in forming physical activity behaviors among Chinese female university students. It extends SCT by revealing complex compensatory and substitutive mechanisms among factors, moving beyond traditional linear models. The findings offer a novel configurational perspective for understanding this population’s physical activity behaviors and provide crucial evidence for designing targeted health behavior interventions.

## 1 Introduction

Physical exercise represents a cornerstone of comprehensive health interventions. The World Health Organization’s Guidelines on Physical Activity and Sedentary Behavior assert that regular physical activity not only improves cardiopulmonary function and metabolic health but also reduces anxiety and depression symptoms, thereby enhancing overall quality of life (1). However, global physical activity participation remains inadequate. Large-scale multinational studies indicate that in 2022, approximately 1.8 billion adults (31.3%) worldwide were insufficiently active (2). This inactivity shows significant gender disparities, with women’s prevalence (33.8%) exceeding men’s (28.7%) by 5.1 percentage points—a gap that has widened persistently over two decades (2).

Data from China’s General Administration of Sport indicate that 37.2% of Chinese people regularly exercised in 2022, remaining substantially below the 2035 target of 45%. Although 55.9% of children and adolescents participate regularly, this rate declines with age (3). Female university students exhibit particularly concerning patterns, with consistently lower participation rates than male students (4). This disparity stems from multiple intersecting barriers specific to this group (5–7). Consequently, the “Healthy China 2030” blueprint identifies women as a key target for physical activity promotion (8).

The university years constitute a critical period for establishing lasting health behaviors. Therefore, understanding the complex causation of physical exercise behavior among female university students carries urgent practical significance. Such understanding is vital for stimulating intrinsic motivation, establishing lifelong activity habits, improving health outcomes, and reducing gender health disparities. Furthermore, it aligns with the educational goals of university physical education and supports the national fitness strategy and population health objectives.

Recent research has significantly advanced our understanding of factors influencing female university students’ physical exercise behavior, examining variables including exercise self-efficacy (9), gender roles (10), motivation (11), and social support (12, 13). However, most studies depend on traditional linear models that isolate “net effects” of individual variables, thereby limiting insights into how multiple conditions interact non-linearly to shape behavior (14). Specifically, linear approaches face two key limitations: First, they struggle to explain causal complexity—particularly whether factors substitute, complement, or reinforce each other. Second, they inadequately address causal equifinality—whereby students with different characteristics achieve similar activity levels through different factor combinations. These methodological constraints hinder deeper mechanistic understanding and consequently limit development of precisely targeted interventions.

Social cognitive theory provides an appropriate framework for this study by emphasizing dynamic, bidirectional interactions among cognition, motivation, and environment—closely aligning with our focus on multifactorial interaction mechanisms. To overcome linear methodological limitations and capture the complex interactions central to SCT, this study employs fsQCA. We develop an “Individual-Behavior-Environment” triadic reciprocity framework to address these core questions: What combinations of personal cognition, motivational drive, and environmental factors produce high physical exercise behavior among Chinese female university students? Can effective configurations be categorized into typical driving patterns? What complementary, substitutive, or transformative mechanisms do these patterns reveal?

## 2 Theoretical perspective and analytical framework

### 2.1 Social cognitive theory (SCT)

Social cognitive theory, developed by Bandura (15), centers on the model of Triadic Reciprocal Determinism. This framework posits that the relationships between the individual (P), behavior (B), and environment (E) form a dynamic, interactive system, rejecting linear causality.

The Individual (P) represents an active agent whose internal cognitions, beliefs, affective states, and physiological characteristics shape behaviors. Behavior (B) encompasses observable actions, including their frequency, intensity, and persistence, reflecting the tangible outcome of individual-environment interactions.

The Environment (E) includes not only physical settings but also social contexts—such as social models, norms, and cultural milieus—that provide conditions, constraints, and opportunities for behavior.

**Figure 1:**
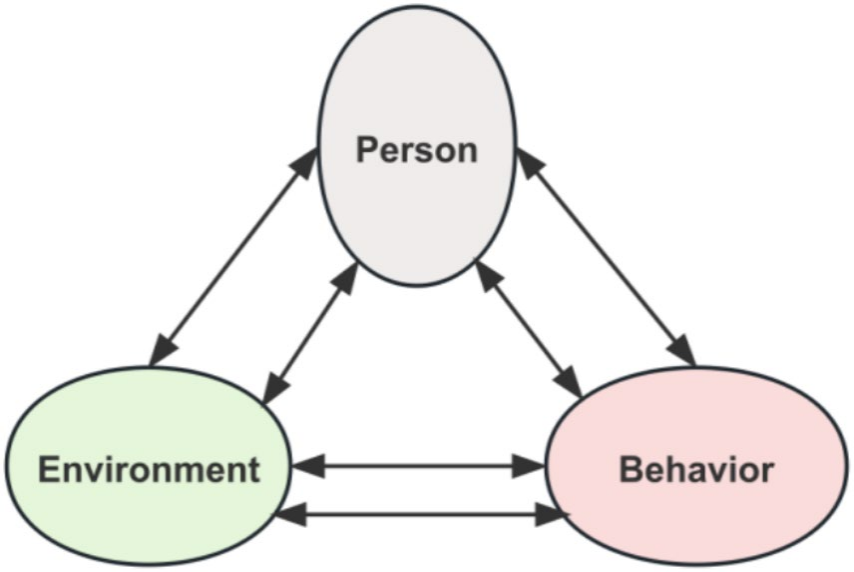
Social cognitive theory model.

Interactions among these elements occur through three bidirectional pathways:

1. P and B: Individual cognitions shape behavior (P → B), while behavioral outcomes, in turn, influence beliefs and affective states (B → P).
2. P and E: Personal characteristics elicit and shape relevant social environments (P → E), while environmental feedback modifies individual cognitions and attitudes (E → P).
3. B and E: Behavior can alter the environment (B → E), and the environment, in turn, constrains or facilitates behavioral expression (E → B) (Figure 1).

The triadic reciprocal determinism model of SCT effectively explains the initiation and maintenance of physical exercise behavior among female university students. Specifically, physical exercise behavior (B) results from interactions between individual factors (P) (e.g., exercise self-efficacy) and environmental factors (E) (e.g., friend support). SCT’s emphasis on dynamic interactions offers a robust theoretical foundation for understanding the formation of this behavior and provides a methodological basis for examining interactive and compensatory effects among influencing factors.

### 2.2 Analytical framework

**Figure 2:**
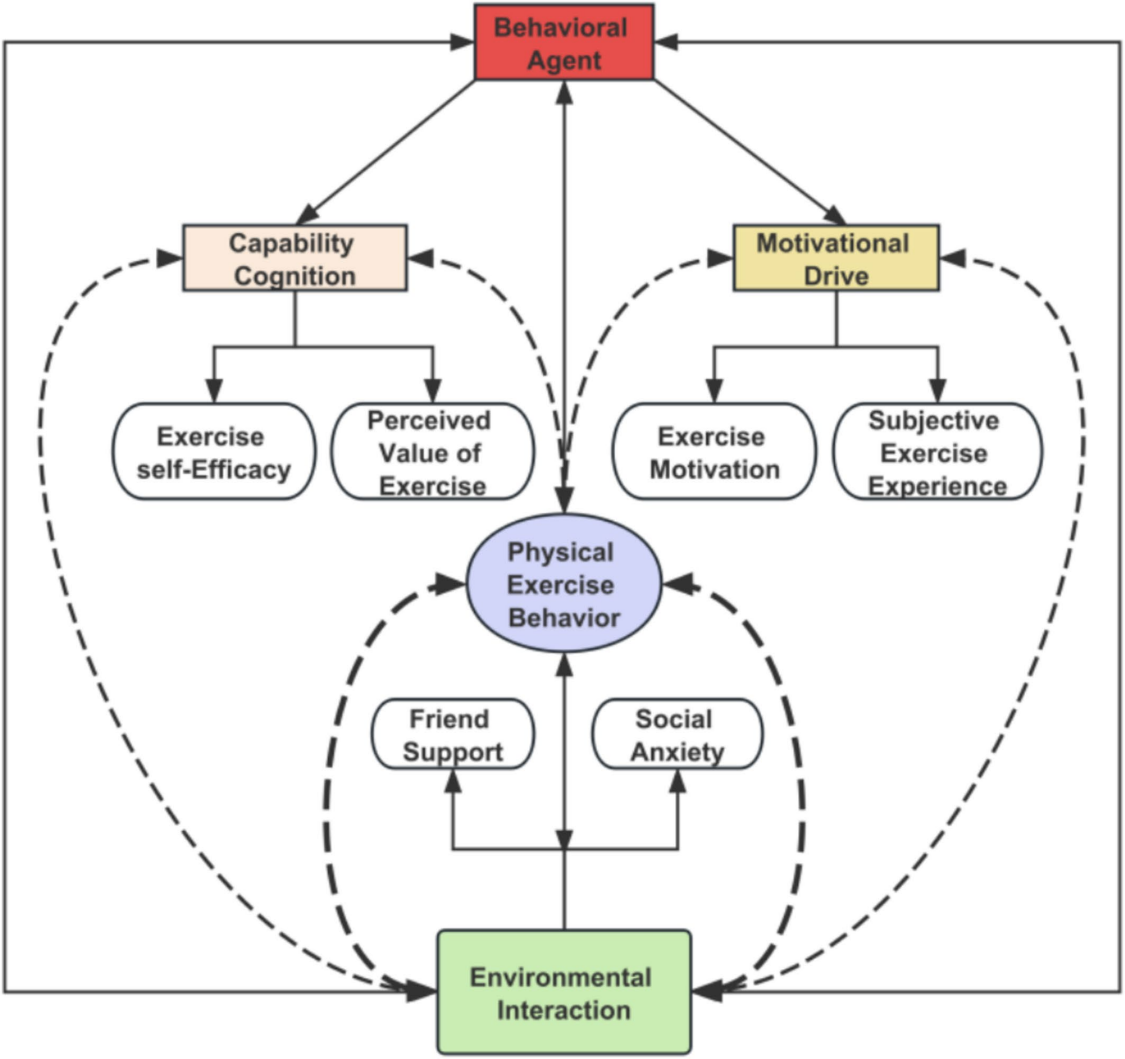
Analytical framework of female university students’ physical exercise behavior from a triadic interaction perspective.

Guided by SCT’s triadic reciprocal determinism model, we constructed an analytical framework for female university students’ physical exercise behavior (Figure 2). First, the framework incorporated the three core SCT dimensions: individual (P), behavior (B), and environment (E), with physical exercise behavior (B) serving as the outcome variable.

We then conducted a systematic literature review to identify relevant condition variables. Using keywords such as “female university students,” “physical exercise,” and “influencing factors,” we searched CNKI, Web of Science, and PubMed, identifying 163 empirical studies published between 2010 and 2025. Inclusion criteria were: (1) focus on Chinese female university students or valid subgroup analyses of this population; (2) examination of factors influencing physical exercise behavior; (3) relevance of condition variables to SCT constructs; and (4) variable occurrence frequency exceeding 50% of retrieved literature (>81 instances) (Table 1).

**Table 1:** Frequency of selected condition variables.

Rank	Variable	Frequency
1	Exercise self-Efficacy ESE	130
2	Subjective Exercise Experience SEE	121
3	Perceived Value of Exercise PVE	108
4	Exercise Motivation EM	103
5	Friend Support FS	98
6	Social Anxiety SA	88
7	Exercise Climate EC	74
8	Self-Esteem SE	43

Following SCT’s core concepts, we selected specific condition variables for each dimension. For the individual (P) dimension, we chose variables representing “capability cognition” (ESE and PVE) and “motivational drive” (EM and SEE). For the environment (E) dimension, we selected FS and SA. These, together with physical exercise behavior as the outcome variable, constitute the analytical framework (Figure 2).

It is important to note that the selected condition variables—particularly those within the individual (P) dimension (ESE, PVE, EM, and SEE)—are theoretically interrelated (e.g., high self-efficacy typically enhances intrinsic motivation). This conceptual overlap is not a limitation but a key strength of the fsQCA approach. FsQCA specifically aims to decipher how theoretically related conditions combine in different configurations to produce equivalent outcomes. These potential overlaps and synergistic effects are central to configurational analysis, embodying the principles of “conjunctural causation” and “multiple pathways.”

## 3 Research method and design

### 3.1 Research method

FsQCA is a methodological approach tailored for small-to-medium sample sizes, integrating both qualitative and quantitative analytical logic. Focusing on the concept of “configurations,” it investigates how combinations of conditions jointly produce specific outcomes, making it particularly suitable for analyzing causal complexity and asymmetric relationships in social phenomena (16). A central objective of fsQCA is to identify multiple equifinal paths—distinct configurations of conditions that lead to the same outcome—where the role of any single condition is interpreted in the context of its interactions with others. We therefore employed fsQCA to analyze the antecedent conditions associated with high levels of physical exercise behavior among female university students. This approach enables the identification of distinct equifinal pathways, overcoming limitations of single-variable analyses and providing micro-level empirical evidence to inform targeted physical activity promotion strategies for this population.

### 3.2 Variable measurement

This study employed an analytical framework consisting of one outcome variable and six condition variables, each measured as follows:

1. Physical exercise behavior (PB)

PB was assessed using the Physical Activity Rating Scale (PARS-3) revised by Liang Deqing (17). This instrument measures exercise volume across three dimensions: intensity, duration, and frequency. Intensity and duration items use a 5-point Likert scale (1–5), while frequency is also rated 1–5. Total exercise volume was calculated as: Intensity × (Duration − 1) × Frequency. The scale showed good internal consistency (Cronbach’s *α* = 0.801).

1. Capability perception dimension

Exercise Self-Efficacy (ESE) was measured using the 10-item scale developed by Kroll et al. (18). Items are rated on a 4-point Likert scale (1 = “completely incorrect” to 4 = “completely correct”), with total scores ranging from 10 to 40. Higher scores indicate stronger self-efficacy. The scale demonstrated good reliability (*α* = 0.858).

Perceived Value of Exercise (PVE) was assessed using the 7-item scale developed by Kirk (19) and adapted by Lu Xinling et al. (20). Responses were recorded on a 5-point Likert scale (1 = “disagree” to 5 = “agree”), with total scores ranging from 7 to 35. Higher scores reflect greater perceived exercise value. The scale showed good reliability and cultural adaptability (*α* = 0.813).

1. Motivational drive dimension

Exercise Motivation (EM) was measured using the shortened MPAM-R scale revised by Chen Shanping et al. (21). All items use a 5-point Likert scale (1 = “not at all” to 5 = “very strongly”), with total scores ranging from 12 to 75. Higher scores indicate stronger motivation to persist in exercise. The scale demonstrated good internal consistency (*α* = 0.895).

Subjective Exercise Experience (SEE) was assessed using the scale developed by McAuley and Courneya (22). Items are scored on a 7-point scale (1 = “never” to 7 = “always”), with total scores ranging from 12 to 84. Higher scores reflect more positive subjective experiences during exercise. The scale exhibited good reliability (*α* = 0.866).

1. Environmental interaction dimension

Friend Support (FS) was measured using the scale developed by Cheng Hui (23). Items are rated on a 5-point Likert scale (1 = “never” to 5 = “always”), with total scores ranging from 5 to 60. Higher scores indicate greater perceived friend support for physical exercise. The scale showed good internal consistency (*α* = 0.813).

Social Anxiety (SA) was assessed using the Interaction Anxiousness Scale developed by Leary and translated by Peng Caizhu (24). The 15-item scale uses a 5-point Likert scale (1 = “strongly disagree” to 5 = “strongly agree”), with total scores ranging from 15 to 75. Higher scores indicate greater social anxiety. The scale demonstrated good reliability (*α* = 0.801).

### 3.3 Ethical considerations

This study received ethical approval from the Institutional Review Board of Jiangxi Normal University (IRB-JXNU-PEC-2025016) and adhered to the principles of the Declaration of Helsinki. Prior to data collection, researchers explained the study purpose, procedures, voluntary participation, confidentiality measures, and potential minor risks to all potential participants. Written informed consent was obtained after ensuring participants’ full understanding and addressing any questions.

The questionnaires were anonymous, and all data were used exclusively for aggregate statistical analysis. No personally identifiable information was collected or disclosed. Throughout data processing and analysis, all data remained anonymized to protect participant privacy.

### 3.4 Data source

This cross-sectional study recruited non-sports major female students from 12 full-time undergraduate universities across China. Data were collected via structured questionnaires administered in a single session between May 6 and 17, 2025, to investigate factors influencing physical exercise behavior.

Sampling followed established QCA case selection principles, ensuring diversity across geographical regions (North, South, Central, East, West) and all 12 academic disciplines. All participants were non-sports major female students, meeting homogeneity requirements. The selected universities represented specialized institutions with distinct academic profiles, providing typical cases. The final sample was evenly distributed across academic years, with rigorous data checks ensuring case accessibility and analytical validity.

We distributed 140 questionnaires using stratified cluster sampling, all returned on-site. We excluded questionnaires with: (1) over 20% missing items; (2) identical responses for more than 70% of items; (3) standardized *Z*-scores beyond ±3 on any dimension. This yielded 115 valid questionnaires, representing an 82.1% valid response rate. Table 2 details the sample composition.

**Table 2:** Demographic profile of the study sample.

Region	Number of universities	Sample size per university	Freshman	Sophomore	Junior	Senior	Total (n)	Majors
Guangdong	4	10	10	10	10	10	40	Philosophy, Economics, Medicine, Agriculture
Hubei	2	15	8	8	7	7	30	Literature, Science
Shandong	2	15	7	7	8	8	30	History, Art
Shanghai	1	10	2	2	3	3	10	Education
Guizhou	1	10	3	3	2	2	10	Management
Liaoning	1	10	2	2	3	3	10	Law
Sichuan	1	10	3	3	2	2	10	Engineering
Total	12	—	35	35	35	35	140	Covering 12 major disciplines

## 4 Empirical results and analysis

### 4.1 Variable calibration

FsQCA employs Boolean logic to examine how combinations of independent variables jointly explain an outcome. Prior to analysis, variables require calibration—transforming raw data into fuzzy sets with values ranging from 0 to 1, representing degrees of set membership (25).

Calibration can be performed indirectly, with researchers setting thresholds based on data characteristics and prior literature, or directly, using software functions for automated transformation. We utilized the direct method in fsQCA software version 4.1, following established practice (26) for its superior objectivity and replicability. Informed by both methodological conventions and the specific context of our research, the calibration thresholds were defined as follows: 95% for full membership, 50% for the crossover point, and 5% for full non-membership (27). A detailed presentation of these thresholds is provided in Table 3.

**Table 3:** Calibration thresholds for condition and outcome variables.

Variable type	Variable name	Full membership	Crossover point	Full non-membership
condition Variables	FS	23.2	17	10.8
	ESE	29	20	12
	PVE	30	25	17
	EM	73.2	53	27
	SA	64.4	47	37.8
	SEE	40	29	17
Outcome	PB	25	18	8

### 4.2 Analysis of necessary conditions

Necessary condition analysis, assessed through consistency indices, represents a preliminary step in configurational analysis. Following established criteria (28), we considered condition variables necessary only if their consistency exceeded 0.9. As shown in Table 4, none of the individual condition variables—FS, ESE, PVE, EM, SA, or SEE—met this threshold for either high or low PB levels.

**Table 4:** Necessity analysis of single antecedent conditions.

Condition Variable	High PB		Low PB	
	Consistency	Coverage	Consistency	Coverage
FS	0.74843	0.746635	0.599564	0.566509
~FS	0.565466	0.598545	0.731851	0.733713
ESE	0.768189	0.800021	0.537031	0.529721
~ESE	0.548433	0.555697	0.797261	0.765119
PVE	0.755405	0.794271	0.558376	0.55607
~PVE	0.577793	0.580071	0.793418	0.754441
EM	0.736239	0.781043	0.557911	0.560577
~EM	0.585784	0.583158	0.782084	0.737422
SA	0.634971	0.671598	0.67284	0.674033
~SA	0.69181	0.690652	0.672178	0.635581
SEE	0.70947	0.785918	0.545308	0.572136
~SEE	0.613755	0.587656	0.795956	0.721824

“~” Indicates the absence or low level of the condition.

This indicates that no single variable is necessary for physical exercise behavior among female university students. Instead, PB emerges from the interactive effects of multiple conditions, warranting further configurational analysis.

### 4.3 Configurational path analysis

#### 4.3.1 Configurational path results

In fsQCA, a configuration refers to a specific combination of antecedent conditions associated with a particular outcome (29). Configurational analysis aims to identify sufficient relationships between condition combinations and outcomes, revealing causal pathways. We established thresholds of 0.8 for consistency, 0.7 for PRI consistency, and 1 for case frequency. Using these parameters, we computed complex, intermediate, and parsimonious solutions, identifying eight configurational pathways leading to high PB (Table 5).

**Table 5:** Configuration paths for high physical exercise behavior.

Condition Variable	Immersive Enjoyment Type		Autonomous Drive Type		Dual Core Reinforcement Type		Efficacy Anxiety Compensation Type	
	Config 1	Config 2	Config 3	Config 4	Config 5	Config 6	Config 7	Config 8
FS				⊗	●	●	●	●
ESE	●	●	●	●	●	●	●	⊗
PVE	〇		〇	⊗		⊗		
EM		⊗	●	●		⊗	●	●
SA		⊗	〇	⊗	⊗		〇	╳
SEE	●	●			⊗	⊗	●	●
Consistency	0.928657	0.918236	0.950725	0.954543	0.924125	0.923374	0.96476	0.941743
Coverage	0.525423	0.357478	0.422072	0.274123	0.402127	0.344407	0.361558	0.331183
Unique Coverage	0.0676588	0.00846577	0.0127326	0.00524879	0.0249233	0.00966793	0.00270909	0.0491186
Solution Consistency	0.88791							
Solution Coverage	0.722118							

● = core condition present; ╳ = core condition absent; 〇 = peripheral condition present; ⊗ = peripheral condition absent; blank = condition irrelevant.

The analysis identified eight distinct causal pathways for high PB, with an overall solution consistency of 0.888 and coverage of 0.722. All individual configurations exceeded the 0.8 consistency threshold, demonstrating strong model robustness (30). These eight pathways share common core conditions and can be categorized into four distinct types.

1. Immersive enjoyment type

This type shares high ESE and positive SEE as core conditions, indicating that intrinsic ability beliefs and immediate emotional rewards are sufficient to drive high levels of physical exercise behavior. Beyond this core, different peripheral conditions form two equifinal pathways.

Configuration 1 (ESE•SEE•PVE) demonstrates that when a high PVE acts as a peripheral condition, individuals’ rational valuation of exercise—such as recognizing its health benefits—synergizes with efficacy beliefs and affective experience (31). This creates a pathway characterized by high integration of “cognition–affect–value,” where behavior is jointly driven by internalized value recognition and immediate pleasure.

Configuration 2 (ESE•SEE• ~ EM• ~ SA) reveals a key insight: even in the absence of explicit EM (~EM), such as specific fitness goals, high exercise behavior can be sustained as long as high ESE and positive SEE are present within a low-social-anxiety (~SA) and permissive environment. This underscores the central role of intrinsic reward mechanisms: confidence in one’s ability (“I can do it”) and enjoyment derived from the activity itself (“I enjoy it”) constitute a powerful internal drive. This form of motivation, arising from the activity’s inherent interest and sense of mastery, can compensate for the lack of externally oriented goal motivation (32). Simultaneously, the low-anxiety environment ensures that this intrinsic drive remains unhindered by external evaluative pressure.

1. Autonomous drive type

This type is jointly driven by high ESE and high EM, forming an “autonomous engine” centered on clear goals and strong ability beliefs. This pattern highlights the potential of the intrinsic motivational system to substitute for insufficient external support.

Configuration 3 (ESE•EM•PVE•SA) shows that when individuals possess strong intrinsic drive (high ESE, high EM) and PVE, they can effectively buffer or even counteract the negative impact of SA. Substantial personal resources provide the psychological resilience needed to cope with negative social evaluations, enabling the maintenance of PB even in high-anxiety situations.

*Configuration 4 (ESE•EM• ~ FS• ~ PVE• ~ SA) offers the most revealing insight: strong ESE and EM can fully compensate for the absence of external support (~FS) and value recognition (~PVE). Provided the social environment is permissive (~SA), an individual’s potent goal-directed willpower (EM) and ESE are sufficient to overcome multiple barriers and independently sustain high levels of PB. This strongly exemplifies the decisive role of “human agency” as conceptualized in SCT.

1. Dual core reinforcement type

This type combines high FS and high ESE as core conditions, reflecting the joint reinforcing capacity of external resources and internal beliefs. The core mechanism of this pattern lies in the ability of external support to effectively compensate for deficits in multiple internal resources.

Configuration 5 (FS•ESE• ~ SEE• ~ SA) indicates that even when SEE is unfavorable (~SEE), encouragement, support, and companionship from friends (FS), combined with an individual’s ESE in a low-anxiety environment (~SA), are sufficient to counteract negative feelings encountered during exercise (33). FS provides an external rationale for persistence and emotional comfort, enabling individuals to transcend temporary physical or affective discomfort and enhancing exercise adherence.

Configuration 6 (FS•ESE• ~ EVP• ~ EM• ~ SEE) represents the pathway with the broadest compensatory scope. When individuals lack internal drive (~EM, ~EVP) and positive subjective exercise experience (~SEE), strong FS and ESE form a powerful “protective buffer.” The external support system directly supplies opportunities for behavior initiation and continuity, while intrinsic self-efficacy ensures the individual’s capacity to utilize this support, thereby overcoming multiple internal deficiencies. This underscores the central role of FS and ESE as protective factors in mitigating complex adverse conditions (34).

1. Efficacy anxiety compensation type

This type shares FS, EM, and positive SEE as common core conditions, forming a stable triangle of “external support–internal drive–process reward.” Within this framework, ESE and SA exhibit a dynamic compensatory relationship: high ESE buffers the negative impact of anxiety, while low ESE requires compensation from a low-anxiety environment. This illustrates the complex interactive mechanisms of resource synergy and substitution within SCT.

Configuration 7 (FS•SEE•EM•SEE•SA) reveals a “spillover effect” of multiple resources. When external support, internal drive, positive experience, and ability beliefs are all at high levels, the resulting “resource redundancy” is sufficient to overwhelm and neutralize the negative effects of SA. PB is thus initiated and sustained through multi-layered, compound motivation.

Configuration 8 (FS•SEE•EM• ~ ESE• ~ SA) demonstrates a compensatory mechanism in which environment and motivation offset low ESE. When an individual’s self-efficacy is low (~ESE), a supportive, low-social-anxiety environment (~SA) becomes the critical compensating factor. In this setting, FS and EM can function without interference, while positive experience (SEE) enhances the appeal of exercise. Together, these factors compensate for the ESE deficit, driving high PB. This underscores the importance of fostering a low-anxiety setting, strengthening external support and internal motivation, and optimizing exercise experiences when ESE is insufficient (35).

#### 4.3.2 Characteristics of configurational pathways

Our findings confirm the complex, context-dependent nature of PB mechanisms (29). Multiple conjunctural causation indicates that no single factor unilaterally determines PB; instead, factors interact through complementary, substitutive, and compensatory relationships (36).

1. Multiple equifinality

All eight pathways effectively lead to high PB, yet their condition combinations differ significantly—involving between two and five core or peripheral conditions—demonstrating clear multiple equifinality in behavioral formation. This implies that female university students with different characteristics and in varying contexts can achieve similar exercise adherence through distinct combinations of factors (37). Such a finding challenges the traditional search for a “universal optimal solution” and underscores the need for more individualized and context-adaptive intervention strategies.

1. Stability of core conditions and causal asymmetry

Our analysis reveals two key insights. On one hand, ESE appears as a core condition in the vast majority of pathways (seven out of eight), highlighting its fundamental and often non-substitutable role—closely aligning with the theoretical core of SCT. On the other hand, the same factor (e.g., PVE, EM, SA, SEE) may appear at high or low levels—or even be absent—across different pathways while still forming part of an effective configuration, a phenomenon known as “causal asymmetry.” This clearly illustrates that a factor’s role depends on its specific combination with other conditions in a given pathway (38). For instance, SA and EM assume different states (present/absent, high/low) in Configuration 2 (ESE•SEE• ~ EM• ~ SA) versus Configuration 3 (ESE•EM•EVP•SA).

1. Compensatory and substitutive mechanisms

The results clearly reveal multiple compensatory and substitutive mechanisms: Intrinsic compensation: ESE and positive SEE compensate for low motivation (Configuration 2). Intrinsic substitution: Strong EM and ESE substitute for the absence of external support and value recognition (Configuration 4). Intrinsic-extrinsic compensation: FS and ESE compensate for multiple internal barriers—low perceived value, weak motivation, and negative experience (Configuration 6). Multi-resource compensation: FS, EM, positive SEE, and a low-anxiety environment collectively compensate for low ESE (Configuration 8). Resource buffering: Multiple positive resources—FS, ESE, EM, and positive SEE—collectively buffer the negative impact of SA (Configuration 7).

1. Conditional transformation of “Negative Factors.”

The most theoretically challenging finding lies in revealing that certain “risk factors” or “negative factors,” consistently validated in linear models, do not exert absolute effects from a configurational perspective but instead exhibit marked context-dependency. Consider SA: traditional research consistently reports a stable negative correlation with PB (39, 40). However, our configurational analysis shows that when individuals possess strong internal resources (e.g., high ESE, EM, and EVP in Configuration 3) or ample external support and positive experience (as in Configuration 7), the negative impact of high SA is effectively neutralized. Its status as a risk factor undergoes a “conditional transformation.” Similarly, negative SEE (~SEE) in Configurations 5 and 6, and low PVE (~PVE) in Configurations 4 and 6, did not prevent the occurrence of high exercise behavior.

This represents an important theoretical refinement: it demonstrates that factors such as social anxiety and negative experience are not deterministic barriers to behavior—their effects depend heavily on the overall “causal recipe” they form with other conditions. Whether a factor constitutes a “risk” depends not on the factor itself, but on its causal context. This “causal context-dependence,” revealed through fsQCA, overcomes the limitation of traditional linear analyses that treat factor effects in isolation, offering a more nuanced perspective for understanding behavioral complexity.

Accordingly, future health behavior interventions should move beyond a singular focus on “reducing risk factors” toward a systemic strategy of “building supportive configurations to transform risks”—that is, by strengthening protective resources such as efficacy, motivation, and support to alter the conditions under which negative factors operate, thereby enabling more precise and effective health promotion.

### 4.4 Robustness checks

Robustness testing aims to verify the stability of core findings by systematically adjusting key parameters or methodological settings under different reasonable assumptions. Results are considered reliable if they remain consistent across multiple sensitivity analyses (41). Common strategies include modifying sample size, recalibrating thresholds, adjusting consistency thresholds to increase or decrease solution stringency, or revising case frequency criteria.

This study employed consistency threshold adjustment for robustness testing. While keeping other parameters constant—including calibration anchors and case frequency—we raised the consistency threshold from the baseline of 0.80 to 0.85 and 0.90, respectively. The results demonstrated that solution consistency remained between 0.887 and 0.892, the configurational paths—i.e., combinations of core and peripheral conditions—showed no significant structural changes, and coverage fluctuated by less than ±0.015.

These findings confirm that under more stringent consistency standards, the core configuration types—Immersive Enjoyment, Autonomous Drive, Dual Core Reinforcement, and Efficacy Anxiety Compensation—and their internal compensatory mechanisms remained stable, verifying the robustness of the conditional variables’ influence on female university students’ PB. We therefore conclude that the configurational analysis conclusions of this study, grounded in SCT, possess strong statistical validity and methodological reliability.

## 5 Conclusions and implications

### 5.1 Conclusion

This study demonstrates that high PB among female university students aligns with the principles of multiple conjunctural causation and causal asymmetry. No single pathway universally explains this outcome; rather, it arises from multiple antecedent conditions interacting through compensatory or substitutive mechanisms. These findings are consistent with other fsQCA studies in health behavior. For example, Zhang et al. (2023) showed that while high SA combined with high ESE can drive PB, low SA does not necessarily lead to low exercise levels, as other factors (e.g., intrinsic motivation) may compensate (42). Similarly, Kane et al. (2022) identified multiple equifinal pathways for healthy eating behaviors where environmental constraints were offset by strong personal resources (43).

This study advances the explanatory power of SCT in the health behavior domain. Our findings not only confirm the dynamic interactions within the triadic reciprocity model but also reveal asymmetric, nonlinear relationships among these factors. The discovery of causal asymmetry shows that the configurations for high PB are not mere inversions of those for low PB. We propose the concept of “conditional transformation of negative factors”: traditional risk factors like SA and negative SEE can have their negative impact neutralized within specific combinations of supportive resources (e.g., high ESE, strong FS, and high EM). This offers an important theoretical correction to the traditional “net effect” perspective on risk factors. Finally, we demonstrate strong methodological synergy between fsQCA and the SCT framework, providing a robust methodological approach for analyzing complex conjunctural causation in health behaviors.

This study offers micro-level evidence for designing targeted health promotion strategies for female university students. Building on the configurational findings, we emphasize the foundational role of ESE and recommend systematic efforts to enhance female students’ confidence in their physical activity capabilities. Concerted actions should strengthen FS networks, for example, by promoting exercise buddy systems and group physical activities, to compensate for potential deficits in individual motivation or SEE. Furthermore, optimizing SEE is essential to foster “Immersive Enjoyment” type behavior. Educational administrators and sports practitioners should abandon one-size-fits-all interventions in favor of differentiated strategies tailored to female student subgroups with different driving patterns. These specific measures represent concrete steps toward implementing the national sports-education integration strategy, promoting adolescent health development, and advancing the “Healthy China 2030″ blueprint.

### 5.2 Limitations

This study has several limitations. First, it identified configurational pathways to high PB without parallel analysis of low PB mechanisms. Future research could examine low PB as a distinct phenomenon. Comparing the causal asymmetry between high and low PB pathways would offer a more complete understanding of the behavioral mechanisms.

Second, the cross-sectional design and modest sample size (*N* = 115) limit causal inference and generalizability. Future studies could use longitudinal or intervention designs to verify the stability of these patterns and establish causality. In addition, expanding the sample’s geographical and cultural diversity, along with incorporating more environmental variables (e.g., school sports policies, facility availability), would help develop a more comprehensive configurational model. Such work would advance health behavior research from correlation toward causal explanation.

In summary, by combining SCT with configurational analysis, this study empirically confirms multiple conjunctural pathways to PB among Chinese female university students. We emphasize that understanding complex health behaviors requires going beyond single determinants or linear relationships to focus on the combinatorial and systemic effects of various conditions. This configurational perspective not only provides a novel analytical framework for female students’ PB but also offers transferable insights for health behavior promotion across other domains.

### 5.3 Recommendations

Based on the four identified configurational types, we recommend differentiated intervention strategies for distinct subgroups of female university students.

1. For the immersive enjoyment type

This subgroup, characterized by high ESE and positive SEE, should receive interventions that strengthen efficacy beliefs through progressive achievement milestones and enhance SEE through multi-context design. In physical education, implement a progressive skill-advancement model—for example, breaking aerobics into sequenced stages (“basic steps → combination routines → choreography and performance”)—using phased “visual certification” to systematically build self-efficacy. In extracurricular settings, create themed immersive scenarios such as “Starlight Fluorescent Night Runs” with lighting and sound effects or “Anime-Themed Fitness Parties” with role-playing to increase exercise enjoyment through multi-sensory stimulation. Instructors should help students progressively increase exercise volume and master sports skills while providing timely positive feedback to strengthen ESE. Instruction should be individualized, adjusting exercise load and intensity according to each student’s needs while monitoring their SEE. Additionally, enhance university sports facilities as primary exercise venues by developing digital infrastructure that provides immediate visual feedback and dynamic tracking to significantly improve SEE. Together, these measures create a “ability certification–experience enhancement” cycle, achieving the synergy between individual cognition and behavioral experience described in SCT.

1. For the autonomous drive type

This subgroup, defined by high ESE and high EM, benefits from a multi-stakeholder approach that addresses peripheral conditions (including value recognition, low-anxiety environment, SA, and external support) to strengthen their intrinsic drive. At the institutional level, promote interdisciplinary collaboration between Psychology and Sports Science in curriculum design. Improve exercise goal clarity and self-management, and establish sports psychology counseling stations with dedicated spaces and tailored facilities for students experiencing SA. Physical education teachers should use personalized goal-setting for different students, incorporate success-sharing sessions into lessons to reinforce ability beliefs, and use visual feedback to enhance PVE. For extracurricular activities, provide diverse, self-directed options and create “audience-free zones” to reduce social pressure. For those lacking FS, facilitate access to virtual sports communities (44), effectively forming virtual workout partners. These coordinated efforts aim to multi-directionally support sustained PB among female university students in this subgroup.

1. For the dual core reinforcement type

Given the core conditions of high FS and high ESE, alongside potential peripheral conditions such as negative SEE, low PVE, or low EM, we recommend constructing a compensatory system that leverages the synergy between FS and ESE. At the institutional level, universities could implement incentive policies for buddy-system based exercise, supporting groups of exercise partners to enroll in courses together and share credit rewards. In instructional design, teachers should structure classes around progressive collaborative tasks that highlight the important role of exercise partners. For students reporting distinct negative experiences, use positive reinforcement strategies and focus on enhancing class enjoyment. Students should actively cultivate a “support-efficacy cycle” through peer encouragement to jointly build exercise confidence, strengthen PVE, and amplify the multiplier effect of strong FS and high ESE.

1. For the efficacy anxiety compensation type

This subgroup is characterized by a triad of core conditions—FS, EM, and positive SEE—together with a dynamic efficacy–anxiety compensation mechanism. To address their specific needs, universities should establish designated exercise anxiety buffer zones that enable students across all anxiety levels to participate in physical activity within reassuring and accommodating settings. Practical steps may involve staggered scheduling that separates high-intensity courses (e.g., cardio kickboxing, Sanda) from low-pressure options (e.g., Tai Chi), thereby helping align individual anxiety profiles with suitable exercise contexts. Physical education teachers should emphasize the integrated application of curricular resources, systematically weaving in distinct pedagogical components—such as structured FS systems, approaches to maintaining motivation, and techniques for enhancing the SEE—throughout the teaching process. This can be achieved by designing practice tasks around a fixed partner system (e.g., paired resistance band exercises) to foster supportive peer relationships; applying established goal-setting frameworks to break down exercise tasks into manageable sequences; and implementing multi-stage evaluation models for assessing student performance in physical education, offering continuous feedback and cultivating advancement. These coordinated initiatives are intended to ensure the sustained maintenance and reinforcement of PB within this particular student subgroup.

## Data Availability

The original contributions presented in the study are included in the article/supplementary material, further inquiries can be directed to the corresponding author/s.
